# Suberinic Acids as a Potential Feedstock for Polyol Synthesis: Separation and Characterization

**DOI:** 10.3390/polym13244380

**Published:** 2021-12-14

**Authors:** Janis Rizikovs, Daniela Godina, Raimonds Makars, Aigars Paze, Arnis Abolins, Anda Fridrihsone, Kristine Meile, Mikelis Kirpluks

**Affiliations:** Latvian State Institute of Wood Chemistry, Dzerbenes 27, LV-1006 Riga, Latvia; daniela.godina@kki.lv (D.G.); raimonds.makars@kki.lv (R.M.); aigars.paze@kki.lv (A.P.); arnis.abolins@kki.lv (A.A.); anda.fridrihsone@kki.lv (A.F.); kristine.meile@kki.lv (K.M.); mikelis.kirpluks@kki.lv (M.K.)

**Keywords:** birch outer bark, suberinic acids, high functionality polyols, polyurethane materials

## Abstract

Global sustainability challenges prompt the world to modify its strategies and shift from a fossil-fuel-based economy to a bio-resources-based one and to the production of renewable biomass chemicals. Depolymerized suberinic acids (SA) were considered as an alternative resource to develop bio-polyols that can be further used in polyurethane (PU) material production. Birch (*Betula pendula*) outer bark was used as a raw material to obtain the SA, extracted with ethanol, and depolymerized with potassium hydroxide ethanol solution. By acidifying the filtrate to pH 5.0, 3.0, and 1.0 and drying it at 50 °C and 130 °C, 12 different SA potential feedstocks were obtained and characterized using chemical (total phenolics content, solubility in DMSO, acid, hydroxyl, and saponification number) and instrumental analytical methods (GC-MS, SEC-RID, DSC, and FTIR). Several bio-polyols were synthesized from the SA sample acidified to pH 1 and dried at 130 °C. Acid number and hydroxyl number values, the apparent viscosity and moisture content were measured. It was concluded that SA have a high enough saponification and acid value to investigate the polyol synthesis route via the esterification reaction. Moreover, SA had OH groups in their structure, which can be exploited for PU material development. The majority of SA compounds had relatively low molecular weight with <1300 Da that are suited for bio-polyol synthesis applied for rigid PU foam development. The synthesized bio-polyols had high hydroxyl number values necessary for bio-polyols to be used for rigid PU foam production.

## 1. Introduction

The European Union (EU) recognizes that bio-based materials are critical to the creation of a more circular and decarbonized economy as well as the transition from a fossil to a bio-based economy. The development of advanced new materials and technologies for bio-based products is critical as the world faces a growing number of issues, leading to growing public awareness about global sustainability. Knowledge-intensive bioeconomy is one of the focus points in Europe’s development along with circular economy [1]. The potential of the available bio-resources in value-added sectors still needs to be fulfilled by new approaches.

The majority of polyurethane (PU) materials are obtained from non-renewable feedstock, which is not in line with the directions (sustainable and inclusive economic development and circularity) along which the EU intends to further develop in the upcoming decades. The most promising way to introduce sustainable feedstock into rigid PU foam is to replace one of its components—petrochemical polyols—with a bio-based alternative. The global green and bio-based polyol market was valued at USD 2.63 billion in 2015 and is projected to reach USD 4.71 billion by 2021 at a 9.5% annual growth rate [2]. Up to now, plant oils have been considered as an alternative resource for the production of polymeric materials and have been extensively studied. By modifying plant oils, it is possible to obtain a large variety of monomers and polymers [3]. The polyol production from epoxidized vegetable oils is already a well-studied topic. Various epoxidation methods of different plant oils such as soybean, palm, canola, castor, jatropha, wild safflower, and others have been reported [4]. However, with the growing global population, it is challenging to balance the use of agricultural land for feedstock production and for food and feed production. Thus, it is essential to use biomass streams that are waste and essentially have little to no value. There are many industries such as the furniture, veneer and cellulose industries that have a large amount of residual birch bark combusted for energy needs. Therefore, processing of birch bark has a high potential in the concept of the circular bio-economy.

Depolymerized suberinic acids (SA) from birch outer bark can be considered as an alternative resource to develop next-generation non-food origin bio-polyols. More than 20 years ago, it was proven that SA obtained from *Quercus suber* engages in polycondensation reactions with isocyanates, showing that former natural macromolecules behave in a straightforward manner in this specific context [5]. These materials contained both thermoplastic and thermoset topologies because of the variable number of hydroxyl groups of SA [6]. In the plant material, suberin is an aromatic-aliphatic cross-linked polyester, in which SA are cross-linked with aromatic polyphenols [7,8].

The outer bark of most birch species, widespread in northern Europe (*Betula pendula* Roth. and *Betula pubescens* Ehrh.), is rich in pentacyclic lupane-type triterpenes, mainly betulin and lupeol, which can be extracted in good yields (20–40%) from birch outer bark with a great number of various organic solvents [9]. Birch outer bark contains up to 45% (dry basis) of suberin [10]. Therefore, after triterpene extraction, the remaining biomass can still be used for the production of other high-added-value products. Hydrolytic depolymerization of suberin through ester cleavage reactions using basic hydrolysis or *trans*-esterification is mainly used to obtain SA. There are several depolymerization methods: alkaline hydrolysis in water [9] and other polar solvents [10,11,12], alkaline methanolysis [4,6,13] using ionic liquids (cholinium hexanoate) [7,14] as well as through the liquefaction process [7]. After alkaline hydrolytic suberin depolymerization, SA salts are obtained. By acidifying the latter, free acids can be isolated. Depolymerized monomer composition of suberin mainly consists of ω-hydroxyfatty acids, α-, ω-dicarboxylic acids, aliphatic alcohols, and aromatic acids. The overall composition of the constituents depends on the plant material and depolymerization conditions. It is known that ω-hydroxy fatty acids are generally the most abundant group of suberin monomers in the outer bark of *B. pendula* and the main compounds in the depolymerized SA mixture are epoxy and hydroxyl groups containing monomers and oligomers [7]. If more drastic alkaline depolymerization conditions (methanolysis) are used, alkanedioic and hydroxyalkanoic acids are formed [11].

Results from the several mentioned methods suggest that suberin samples were still partially polymerized, or at least in the form of esterified oligomeric structures with molecular weights high enough to hamper their detection by GC–MS. Such oligomeric fractions of SA are also not desirable in obtaining polyol. In this research, alkaline hydrolysis in ethanol was chosen because this process is more scalable, has the potential to obtain more monomeric fractions, and is environmentally friendly. The aim of the research was to evaluate the potential of birch outer bark SA as a raw material for pilot scale polyol synthesis with less oligomeric fractions, and the impact of process conditions on the resulting SA content and properties. Therefore, characterization of physical and chemical properties after separation at different pH levels and drying temperatures for SA and the obtained bio-polyols was carried out.

## 2. Materials and Methods

### 2.1. Raw Material—Birch Outer Bark

Isolated and fractionated birch outer bark from the AS Latvijas Finieris plywood factory (Latvia) was selected as a representative industrial waste. Birch outer bark samples were dried at room temperature (moisture content 4–5%) and milled in a SM 100 cutting mill (Retsch GmbH, Haan, Germany) to pass through a sieve with holes measuring 4 mm in diameter. Milled birch outer bark was fractionated by sieving using an AS 200 Basic vibratory sieve shaker (Retsch GmbH, Haan, Germany); a fraction of 1–3.15 mm was collected. Fractionated birch outer bark was extracted with ethanol twice, as described by Godina et al. [12]. After extraction, birch outer bark with a moisture content of ca. 5–6% was used as a feedstock for depolymerization.

### 2.2. Other Materials and Chemicals

Glacial acetic acid (AcOH), puriss., ≥99.8%; acetanhydride, puriss., ≥99%; diethyleneglycol (DEG), reagent grade, 98%; 4-(dimethylamino)pyridine (DMAP), reagent plus, ≥99%; *N*,*N*-dimethylformamide (DMF), ACS reagent, ≥99.8%, water content ≤150 ppm; hydrochloric acid (HCl), ACS reagent, ≥37%; nitric acid (HNO_3_), for analysis, 69%; sulfuric acid (H_2_SO_4_), ACS reagent, 95.0–98.0%; acetone, ACS reagent, ≥99.5%; tetrahydrofuran (THF), anhydrous, ≥99.9%, inhibitor-free; methanol, ACS reagent, ≥99.5%; pyridine (Py), suitable for HPLC, ≥99.9%; dimethyl sulfoxide (DMSO), ACS reagent, ≥99.9%; 2-propanol (i-PrOH), ACS reagent, ≥99.5%; dichloromethane (DCM), anhydrous, ≥99.8%; potassium hydroxide (KOH), puriss., ≥85%; sodium hydroxide (NaOH), puriss., ≥85%; sodium carbonate (Na_2_CO_3_), puriss., ≥85%; sodium periodate (NaIO_4_), ACS reagent, ≥99.8%; ammonium molybdate (NH_4_)_2_MoO_4_), 99.98% trace metals basis; sodium thiosulfate (Na_2_S_2_O_3_), ReagentPlus^®^, 99%; zinc acetate dihydrate (ZnAc), ACS reagent, ≥98%; potassium iodide (KI), ACS reagent, ≥99.0%; Silylating mixture III, for gas chromatography (GC) derivatization; *Folin–Ciocalteu*’s phenol reagent was ordered from Sigma-Aldrich (Schnelldorf, Germany).

### 2.3. Preparation of SA

**Figure 1:**
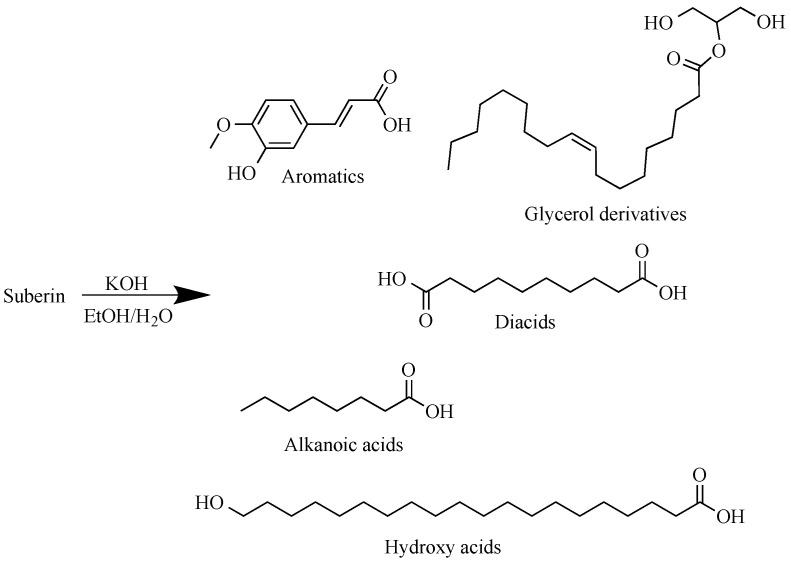
Representative structures of monomeric components resulting from suberin depolymerization.

About 2000 g of dry extracted birch outer bark was depolymerized in potassium hydroxide (650 g) ethanol–water solution (20 L) for 1 h at 80 °C (extracted outer bark–ethanol–water–potassium hydroxide mass ratio 1:7.3:0.73:0.27). Approximate representative structures of monomeric components resulting from suberin depolymerization can be seen in Figure 1.

**Figure 2:**
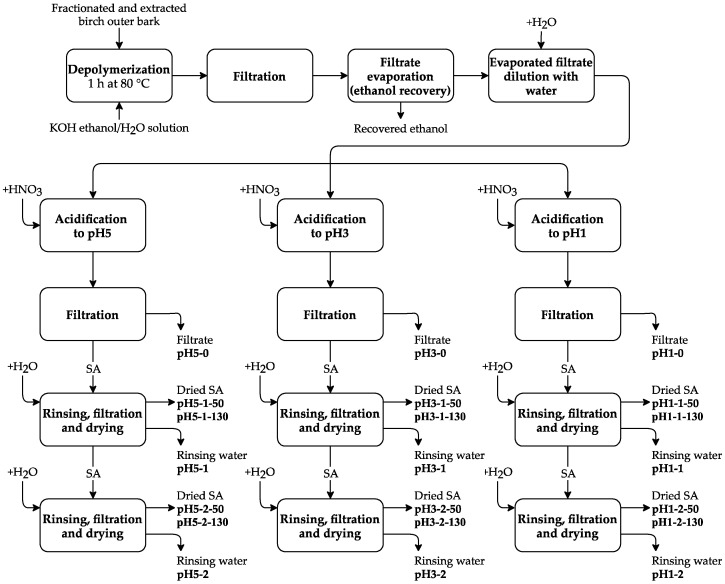
Experimental scheme for obtaining the SA samples.

After depolymerization, the solution was cooled down to 25 °C and filtered. The filtrate was evaporated until 70% of ethanol–water solvent was recovered, followed by further dilution with 4 L of water. The obtained suspension was divided into three equal parts followed by acidification with HNO_3_ to pH 5.0, 3.0, and 1.0. Each pH fraction was filtered and rinsed with deionized water one or two times, followed by filtration and drying at 50 °C or 130 °C for each fraction. Thus, 12 SA fractions were obtained (see Figure 2).

### 2.4. Characterization of SA and Rinsing Water

#### 2.4.1. The Total Phenolics Content (TPC) in Rinsing Water and SA Samples

To about 1 g of the sample, 10 mL of DMSO was added. Glass tubes with the solutions were left at room temperature for 20 h and then placed in an ultrasonic bath for 60 min. The solutions were then filtered through a glass frit filter (pore size 2). The filtrate was diluted 2× for TPC analyses. The filters were washed and dried at 103 °C for 20 h and the amount of solid residue was calculated from the difference in mass. To determine TPC, 7.9 mL of deionized water was added to plastic tubes, 0.1 mL of the diluted filtrate, and 0.5 mL of *Folin–Ciocalteu*’s reagent was added. The solutions were conditioned in the dark for 8 min. Then 1.5 mL of 20% Na_2_CO_3_ solution was added, and after 2 h of incubation in the dark, the absorbance at 756 nm was measured. The TPC in SA rinsing water samples was determined as described previously, but without sample dilution for the analysis. Gallic acid was used as a standard solution (0.05; 0.1; 0.3; 0.6; 0.8; 1.0 mg·mL^−1^) to determine the phenol content as gallic acid equivalents (GAE) mg·g^−1^ and further recalculated as total phenolic content (*TPC*, %) by mass (Equation (1)).

(1) $$TPC=\frac{\frac{A-b}{a}}{10}\times DF$$

*A*—absorption, au;
*a*, *b*—coefficients of gallic acid calibration curve; and
*DF*—dilution factor.

#### 2.4.2. Acid Number

To about 0.2 g of the sample, 5 mL of DMSO was added and stirred for 1 h. Afterward, 20 mL of i-PrOH and 5 mL of water were added, and the solution was titrated with 0.1 M KOH solution. Two replicate experiments were performed for each sample.

#### 2.4.3. Epoxy Groups

To about 0.2 g of the sample, 10 mL of 0.2 M HCl in acetone was added. The solution was stirred for 1 h and titrated with a known concentration of 0.1 M KOH solution.

#### 2.4.4. Saponification Number

To approximately 1.5 to 2.0 g of the sample, 25.0 mL of the reagent (0.5 mol/L KOH in ethanol) was added. The reaction mixture was heated at 60 °C for 30 min. It was then immediately titrated with 0.5 mol/L HCl solution.

#### 2.4.5. Hydroxyl Number

Preparation of the acetylating mixture (10%)—to 90 mL of dry DMF acetic anhydride (10 mL) was added. The solution was stirred and placed overnight in a dark place. Preparation of the catalyst solution (1% DMAP)—2 g of DMAP was dissolved in 200 mL of anhydrous DMF. Assay procedure—to about 0.2 to 0.5 g of the test sample 15 mL of catalyst solution was added, and the sample was completely dissolved. To the dissolved test sample, 5 mL of acetylating mixture was added and stirred with a magnetic stirrer for 15 min. The solution was titrated with 0.5 M KOH solution.

#### 2.4.6. Gas Chromatography-Mass Spectrometry (GC-MS) Analysis

After preparation according to methods 1 or 2, the sample (1 μL) was injected into a Thermo Scientific TRACE 1300 gas chromatograph with a Thermo Scientific ISQ quadrupole mass detector (Waltham, MA, US). A Thermo Scientific TG-5MS (30 m × 0.25 mm × 0.25 µm) column was used. Injector temperature: 250 °C. In splitless mode, carrier gas (helium) flow 1.20 mL/min. Oven temperature program: isothermally held at 150 °C for 5 min, then increased at 10 °C/min and held for 1 min, before being finally increased at 2 °C/min and passed at 300 °C for 15 min. The total time of analysis was 60 min. The transition line temperature of the mass detector was 250 °C, and the ion source temperature was 200 °C. Mass range 45–700 Da. Each sample was analyzed by two complementary methods:

Method 1: SA samples were converted to the corresponding trimethylsilyl (TMS) derivatives and analyzed quantitatively by GC-MS, allowing the identification of monomeric structures present in the mixture. SA samples (approximately 50–100 mg) were reacted with 100 µL of pyridine, 200 µL silylating mixture III (1-(trimethylsilyl) imidazole/BSTFA/TMCS 3/3/2 (*v*/*v*/*v*)) for 20 min at 70 °C.

Method 2: In order to analyze the composition of the oligomeric/polymeric fraction of suberin, samples were, prior to silylation, submitted to alkaline hydrolysis to release hydrolyzable monomeric constituents. Approximately 50–100 mg of SA samples was dissolved in 1 mL of methanol, and then 1 mL of 1 M NaOH in water was added, and samples were incubated at 95 °C for 4 h. The mixture was cooled to room temperature, acidified to pH 3–3.5 with 1 M HCl, extracted three times with DCM, and the combined organic extracts were dried in a rotary evaporator. Finally, samples were trimethylsilylated as above-mentioned prior to GC-MS analysis.

#### 2.4.7. Size-Exclusion Chromatography with Refractive Index Detector (SEC-RID) Analysis

SEC-RID analysis of the SA samples was conducted with Advanced Polymer Chromatography (APC), equipped with APC XT 200 2.5 µm, XT 450 2.5 µm, and XT 45 1.7 µm columns at 40 °C (Waters Acquity, Manchester, UK). An APC refractive index (RI) detector was used. The thermostat temperature for the RI detector was 40 °C. The mobile phase was THF with a flow rate of 0.6 mL/min. SA samples were prepared as THF solutions with 15 mg/mL mass concentration and filtered through nylon syringe filters to remove undissolved solids (0.22 µm). The injection volume was 20 µL. PSS polymer standards were used for calibration with a 3rd order polynomial calibration curve: y = −0.0047x^3^ + 0.058x^2^ − 0.70x + 7.7 (R^2^ = 0.998). Data acquisition and processing were carried out using Empower 3 and Microsoft Excel software.

#### 2.4.8. Differential Scanning Calorimetry (DSC) and Fourier Transform Infrared (FTIR) Analysis

A differential scanning calorimeter DSC822 (Mettler-Toledo, Greifensee, Switzerland) was used to analyze thermal effects in the SA samples. The analysis was performed for the aluminum-encapsulated sample under N_2_ purging at three stages: (1) cooling from 20 °C to −50 °C; (2) heating from −50 °C to 100 °C; and (3) cooling from 100 °C to −50 °C at a rate of 10 °C/min. The SA structure was analyzed by FTIR spectrometry data obtained with a Nicolet iS50 spectrometer (Thermo Fisher Scientific, MA, US) at a resolution of 4 cm^−1^, 32 scans. The FTIR data were collected using the attenuated total reflectance technique with a diamond crystal prism.

#### 2.4.9. Determination of Hexoses in SA Rinsing Water Samples

To about 1–3 g of the rinsing solution was 0.2 mL of 15% H_2_SO_4_ and 1 mL of 0.2 M NaIO_4_ solution added. The obtained solution was immediately placed in the oven at 40 °C. After 4.5 h, the solution was removed from the oven and immediately 5 mL of 10% (NH_4_)_2_MoO_4_ solution was added. After 15 min, 1 mL of AcOH and 1 mL of 10% KI solution were added and after 15 min, titrated with 0.1 M Na_2_S_2_O_3_ solution.

### 2.5. Preparation of Polyols and Their Characterization

**Figure 3:**
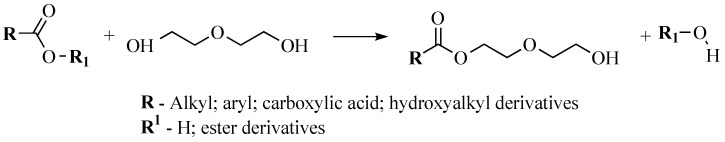
Representative reaction diagram to obtain bio-polyols from the SA.

Taking into account the obtained results from obtaining and characterization of SA, bio-polyols were synthesized from the SA sample, which was acidified to pH 1 and dried at 130 °C via the esterification reaction with DEG. An approximate representative reaction diagram to obtain bio-polyols from the SA can be seen in Figure 3.

The molar amount of the DEG needed for bio-polyol synthesis was calculated using the following Equation (2):

n(DEG) = n(sap. v)(2)

where n(DEG) is the molar amount of the alcohol used for esterification reaction in mol and n(sap. v) is the molar amount of the SA sample saponification value in mol.

**Figure 4:**
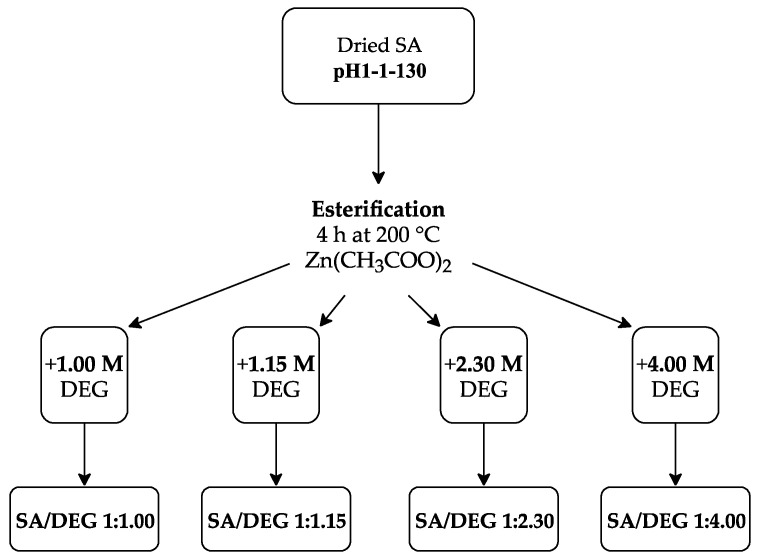
Experimental scheme for obtaining polyols from the SA sample.

Accordingly, the following SA to DEG ratios were used: 1:1.00, 1:1.15, 1:2.30, and 1:4.00. Therefore, the following acronyms were used for each polyol: SA/DEG 1:1.00, SA/DEG 1:1.15, SA/DEG 1:2.30, and SA/DEG 1:4.00 (see Figure 4). The reaction was carried out in a three-neck round bottom flask to which the appropriate mass of SA sample, DEG and ZnAc as a catalyst (0.15 wt.% of SA sample) were added. The flask was put into an oil bath and a mechanical stirrer was inserted into the central neck. A Liebig condenser and a thermocouple were attached to the vacant necks. The mechanical stirrer was set to 300 rpm and the flow of purge gas (N_2_) through the flask was provided, while the content of the flask was heated up to 200 °C. When the required temperature was reached, the stirring of the reaction medium and the N_2_ flow was retained for 4 h for all synthesized polyols.

The obtained bio-polyols were characterized for hydroxyl and acid values according to the analytical methods using titration as described previously for SA characterization in Section 2.4.2 and Section 2.4.5, respectively. The apparent viscosity of polyols was measured at 25 °C, and the shear rate 50 s^−1^ with the MCR 92 rheometer (Anton Paar, Graz, Austria) using standard flow curve measurement and shear rate sweep from 0.1 to 100 s^−1^. The moisture content was measured with the automatic titrator Model 275KF (Denver Instrument, Bohemia, NY, US) using Karl Fisher titration. Polyol structure was analyzed using FTIR, similar to the SA samples as described in Section 2.4.8.

## 3. Results and Discussion

### 3.1. Preparation of SA

Overall, 12 SA fraction samples were obtained. The variables for each fraction were the acidification pH, times of rinsing, and drying temperature. After acidification, filtration, and rinsing of SA, the resulting filtrates and rinsing waters were collected and analyzed. The amounts of total solids, TPC, and hexoses were estimated in the resulting samples and are expressed as a percentage of the filtered (rinsed) component on the dry SA basis. As seen from the results in Table 1, most of the solids ranging from 25 to 29% were present in the filtrate of the SA suspension after acidification. Most of the solids were KNO_3_ formed after acidification of potassium suberinates with HNO_3_. The amount of hexoses filtered (rinsed) was higher for the samples with a higher acidification value. Filtrates from the pH 5 samples had the highest TPC values (0.49%) in comparison with the TPC values in the filtrates (0.21%) obtained after acidification to pH 1, showing that phenolic compounds are precipitated at lower pH.

The dry matter content and yield for the fractions obtained before drying are shown in Table 2, and these results are further discussed in Section 3.2.1.

### 3.2. Chemical Characterization of SA Samples

#### 3.2.1. Chemical Properties of SA Samples

A number of instrumental analyses were carried out for all 12 samples to determine the chemical properties of SA. TPC determination, acid number, saponification number, epoxy groups, and hydroxyl number determination. The results of chemical properties are summarized in Table 2. Although all results obtained were similar, several tendencies were observed. By increasing the pH value of SA acidification from pH 1 to pH 5, TPC in the obtained samples slightly decreased from 3.57–4.00 at pH 1 to 2.19–3.00 at pH 5, indicating that phenolic compounds tend to precipitate at more acidic conditions. This trend was also confirmed by the results of the SA rinsing filtrates, where filtrates from pH 5 samples had higher TPC values in comparison with filtrates from pH 1 samples (Table 1). A similar trend showed a decrease in the saponification number from 2.85–3.72 mmol/g at pH 1 to 2.37–2.87 mmol/g at pH 5, whereas hydroxyl number showed the opposite trend—increasing from 3.64–4.09 mmol/g at pH 1 to 3.97–4.64 mmol/g at pH 5. Saponification and acid values are important for polyol synthesis because these values show the potential of how much the according sample can be esterified. From the technological point of view, it is desirable that these values are not temperature sensitive.

When sample drying temperature was increased from 50 °C to 130 °C, the acid number and epoxy group content slightly decreased, indicating that samples are taking part in condensation reactions, thus forming new intermolecular structures at elevated temperatures.

Dry matter content was higher at lower acidification pH, whereas the yield showed the opposite trend (see Table 2). The times of rinsing did not have a significant effect on the dry matter values. The yields for all six fractions were calculated on the dry basis of one-sixth part of the initial feedstock—dry extracted birch outer bark. The higher yields at higher pH values can be explained by the consistency of the paste-like SA, which, after acidification, were stickier and more swollen. Additionally, it was difficult to rinse and separate SA by filtration. Therefore, part of potassium suberinates was left in the SA paste, which resulted in an increased product yield. The decrease in dry matter content with an increase in pH also supported this observation. SA obtained after acidification to pH 1 were less consolidated and therefore about two times faster to separate from the suspension after rinsing and acidification. Higher dry matter value is important for saving water and energy resources.

When considering the sample rinsing procedure, there were no noticeable differences when comparing samples rinsed one or two times, so sample count for further instrumental analyses (GC-MS, SEC-RID, DSC, and FTIR) was decreased to six by characterization of the samples that were rinsed only once.

#### 3.2.2. Instrumental Analysis of SA Samples

The results of the GC-MS analysis of SA samples are shown in Table 3. Two methods were performed—direct SA silylating (Method 1) and depolymerization with NaOH solution and silylation pre GC-MS analysis (Method 2). According to GC-MS data, pH had a relatively little effect on the relative composition of the SA. By comparing Methods 1 and 2, it can be concluded that after ethanol alkaline hydrolysis, mostly hydroxy acids were separated from the suberin macromolecule because their relative amount predominated in the samples. Similar results were obtained by Ferreira et al. [8], where ionic liquids were used for depolymerization. In comparison, our method using ethanol alkaline hydrolysis suberin was depolymerized to a higher extent because after Method 1, the relative amount of hydroxy acids was even more than 10 times higher (29.16–45.54 rerl%) than in the case of ionic liquids. Results from Method 1 showed that only at pH 1, the relative amount of hydroxy acids increased at higher drying temperature, but at pH 3 and pH 5, the opposite trend was observed. Increased temperature and pH value also affected the relative amount of diacids. After Method 1, only at pH 3, pH 5, and drying temperature, 50 °C decanedioic acid was present, while at pH 1, it was not detected. The relative amount of diacids changed significantly with increasing acidification pH at drying temperature of 50 °C (pH 1–10.48 rel% → pH 3–17.67 rel% → pH 5–23.27 rel%). At a drying temperature of 130 °C, the opposite trend was observed (pH 1–9.66 rel% → pH 3–6.96 rel% → pH 5–5.00 rel%). Thus, it can be concluded that SA precipitated at higher pH are less resistant to higher drying temperatures.

It can be seen that betulin after Method 2 could not be detected or the relative amount of betulin was below limit of detection (Table 3). This can be explained by the fact that by performing depolymerization with NaOH solution (Method 2), betulin formed a precipitate. At pH 5 and 130 °C, the relative area% of lupeol was larger than for other samples, so it was possible that the sample used for GC analysis was inhomogeneous. Samples without monomeric fraction also contained a significant amount of the polymeric suberin fraction, which cannot be seen by GC-MS without hydrolysis. Therefore, it is important to perform sample analysis according to these two methods.

At pH 5, the relative amount of aromatic compounds decreased with increasing SA drying temperature. This could be explained by the esterification of tannins with SA, resulting in such a copolymer that GC-MS cannot determine. Therefore, the content of tannins, hydroxy acids, and diacids decreased and the content of extractives increased because other compounds were relatively less; these were not determined.

**Figure 5:**
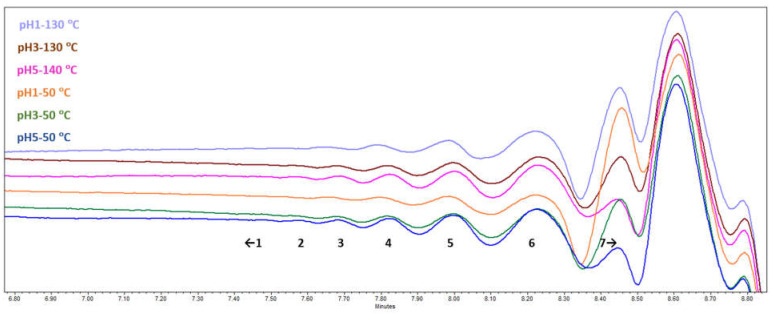
SA sample SEC-RID chromatograms.

During the SEC-RID analysis (Figure 5), seven fractions with different molecular weights could be resolved. Traces of the largest molecules with molecular weight >5500 Da were detected only in the pH 5 samples, while the following overall trend was that the lower the molecular weight of a resolved fraction, the bigger its relative abundance. However, it is possible that the highest molecular weight components were left out during the sample preparation stage before SEC-RID, as only partial solubility in THF at the given concentration was observed. Comparative fraction distribution showed that bundles of oligomers could definitely be seen, which means that depolymerization had taken place, and the effect of acidification pH and drying temperature on the oligomeric fractions of the SA obtained could be estimated.

For molecular weight <1300 Da (peak 7 in Figure 5 and Table 4), the results were outside the calibration scope and could not be more adequately estimated. When comparing different acidification pH, it can be seen that as the pH increased, the lower molecular weight fraction (<1300 Da) slightly decreased from 77 rel% at pH 1 to 68 rel% at pH 5 and the relative areas of the higher molecular weight fractions increased.

When comparing different drying temperatures, it can be seen that there was no significant trend. Only SA samples after acidification to pH 5 showed a more pronounced tendency for the relative area of small fractions to increase and higher fractions to decrease as the drying temperature increases. This confirmed that samples (pH 5) are thermally less stable and at higher drying temperatures, are undergoing some further depolymerization or recombination reactions. To approve such an assumption, DSC analysis was carried out.

**Figure 6:**
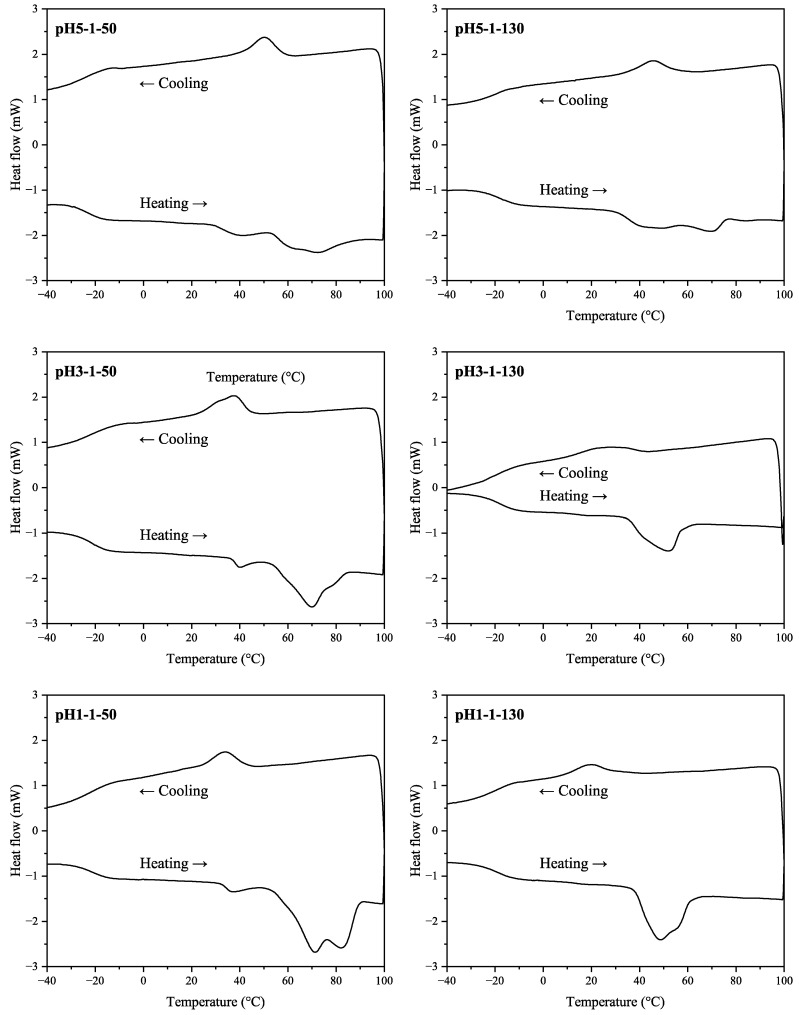
DSC thermograms of the SA samples.

DSC thermograms for SA samples from the 2nd stage (heating) and the 3rd stage (cooling) are shown in Figure 6. Depending on the sample, two or three melting peaks were observed (including hidden peaks for samples pH3-1-130, pH1-1-50, pH1-1-130). The peak values are given in Table 5 including T_g_ and T_c_ values.

For the samples dried at 50 °C, there was a tendency for the T_m2_ and T_m3_ to shift at higher values with a decrease in pH. For the pH1-1-50 sample, the melting area was most distinguishable compared to samples with higher pH values. Thus, at lower acidification pH, more crystalline SA fractions with higher melting points were formed. In the cooling stage, the acidification pH also affected the crystallization pattern. For both SA drying temperatures, the crystallization temperature decreased with a decrease in pH. The acidification pH did not affect T_g_ values, however, these values were higher for samples dried at higher temperatures ranging from −19.6 to −17.9 °C.

Overall, elevated sample drying temperature had a higher impact on samples with lower acidification pH values. When comparing pH5-1-50 and pH5-1-130 samples, there were minor changes in the DSC pattern. Additionally, there was virtually no difference in acid number and epoxy group values in the corresponding samples (see Table 2). The most noticeable difference appeared at pH 1, where for sample pH1-1-50, there were two major melting peaks at approximately 71 and 82 °C, whereas sample pH1-1-130 had two overlapping melting peaks at 48 and 56 °C.

Overall, the melting areas were smaller for the samples dried at elevated temperatures. This behavior could be explained by potential condensation reactions that have occurred, resulting in larger molecules that are less prone to crystallization and lower melting points. Possible group condensation products could be tannins esterified with SA. For example, quebracho bark tannins esterified with stearic acid melt at around 55 °C [13], which complies with data for samples pH3-1-130 and pH1-1-130. However, data from GC-MS analysis in Table 3 shows that pH 1 sample had a higher hydroxy acid relative amount when dried at a higher temperature. This behavior, in contrast, suggests that further SA depolymerization occurred in the sample while drying. Overall, we propose that simultaneous condensation and depolymerization reactions took place at elevated temperatures (130 °C) for all samples. Still, the trend seems to be that for the more acidic samples, the depolymerization route dominated, while with higher pH—the condensation route.

**Figure 7:**
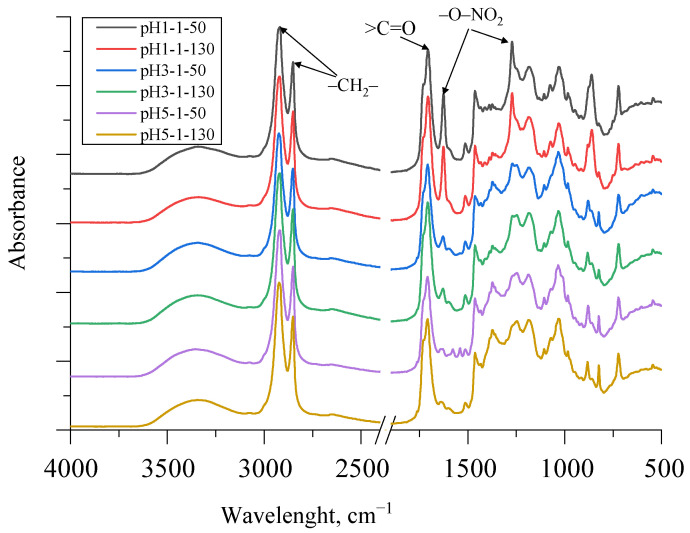
FTIR spectra of SA samples using different acidification and drying strategies.

FTIR spectra of SA obtained using different acidification strategies as well as different drying temperatures are depicted in Figure 7.

There was a very strong peak at 1710 cm^−1^, representing carboxylic acid groups, in all SA samples of different fractions with a trend that the peaks were higher at lower pH. This was a typical stretching vibration of the >C=O group. A small shoulder of this peak also appeared at ~1730 cm^−1^ as the SA sample contained some esters, which also corresponded to the titrated saponification number in Table 2. The peaks at 1463 cm^−1^ and 1375 cm^−1^ indicate asymmetric and symmetric C–H deformations of the aliphatic regions, which may be characteristic of suberin [14,15]. As can be seen in Figure 7, the SA fraction, which was acidified to pH 1, contained a noticeable residue of HNO_3_. The symmetric and asymmetric stretching vibrations of –O–NO_2_ at 1270 cm^−1^ and 1625 cm^−1^ can be seen in the spectra. However, as the pH value increased, the peaks at 1270 cm^−1^ and 1625 cm^−1^ decreased significantly. Additionally, symmetric and asymmetric –CH_2_– stretching peaks were observed at ~2930 and 2855 cm^−1^ for SA samples. The broad absorption peak between 3600 and 3100 cm^−1^ of all spectra of the obtained SA fractions was identified as characteristic stretching vibrations of the –O–H group.

### 3.3. Preparation and Characerization of Bio-Polyols

All four bio-polyols were prepared from the SA fraction that was acidified to pH 1, rinsed once, and dried at 130 °C. The sample was chosen based on the obtained results that at lower acidification pH, a higher amount of low molecular fractions was obtained. Higher drying temperature did not affect the properties significantly when compared to that at 50 °C. At 130 °C, SA is in a melted state, when it is possible to feed them in the reactor when thinking about the future scale-up. The acid value and saponification value of this fraction was 1.56 ± 0.03 mmol/g (87 ± 2 mg KOH/g) and 2.85 ± 0.07 mmol/g (159 ± 4 mg KOH/g), respectively. Polyols were synthesized using four different molar ratios of the esterification reactant DEG. The increase in molar ratios between SA fraction and DEG led to a better esterification reaction as the acid value decreased gradually. However, by increasing molar ratios, there was a noticeable increase in the hydroxyl value, which could be explained by the increased amount of DEG in the reaction mixture.

The apparent viscosity followed a similar trend as the acid value, but opposite to the hydroxyl value. This could be explained by the increased molar ratios between SA fraction and DEG. The moisture of the samples was in the range from 0.145 to 0.455% (Table 6). The obtained bio-polyols from SA and DEG could be used in rigid PU foam development. However, in future, if the developed SA bio-polyols would be used in the production of rigid PU foams, further studies need to be conducted to maintain low ratios between SA fraction and DEG, but at the same time achieve lower acid value as well as a decreased apparent viscosity.

The key characteristics of the synthesized bio-polyols such as acid value, hydroxyl value, apparent viscosity, and moisture content are summarized in Table 6.

**Figure 8:**
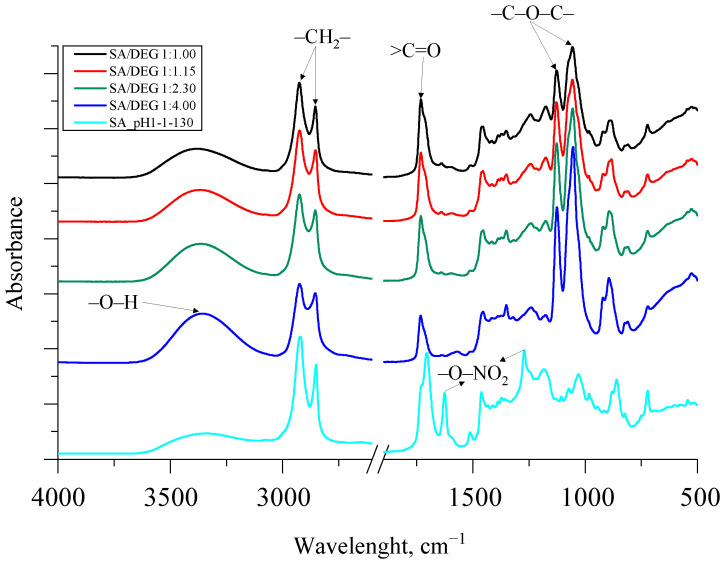
The FTIR spectra of the synthesized SA polyols.

The synthesized bio-polyols had high hydroxyl values, which is typical and necessary for polyols to be used for rigid PU foam production. Unfortunately, the bio-polyols reached very high viscosity, which can be explained by a very high molecular weight of the bio-polyols. The FTIR spectra of the synthesized SA bio-polyols are depicted in Figure 8.

In contrast to SA, for all the synthesized polyols, symmetric and asymmetric stretching vibration of –O–NO_2_ at 1270 cm^−1^ and 1625 cm^−1^ in the spectra disappeared, which was because of the evaporation of nitric acid from the reaction mixture. As for the obtained SA/DEG polyols, the typical stretching vibration of alkoxy groups (ether groups) was visible at 1070 and 1130 cm^−1^. The intensity of these peaks corresponded with the amount of added DEG. The higher the amount of the added DEG, the higher intensity of peaks.

Furthermore, the esterification reaction between SA and DEG was noticeable because of the bathochromic shift of a carbonyl group >C=O from 1710 cm^−1^ to 1730 cm^−1^. Additionally, symmetric and asymmetric –CH_2_– stretching peaks were observed at ~2930 and 2855 cm^−1^ for the SA sample and all the obtained bio-polyols from SA and DEG. The broad absorption peak between 3600 and 3100 cm^−1^ in all spectra of the synthesized bio-polyols was identified as characteristic stretching vibrations of the –O–H group. The intensity of the peak between 3600 and 3100 cm^−1^ of the synthesized SA/DEG polyols and the SA sample correlated with the increase in the hydroxyl value obtained from the titrimetric analysis of the polyols presented in Table 6.

## 4. Conclusions

SA have a high potential in bio-polyol production. They have a high value of saponification, hydroxyl groups, and acids, which can be esterified. The increase in the pH value of SA acidification from pH 1 to pH 5 slightly decreased the saponification number in the obtained samples, whereas the hydroxyl number showed the opposite trend. These values were not temperature sensitive, which from the technological point of view is very desirable.

In GC-MS analysis, it was concluded that after depolymerization, all samples were dominated by hydroxy acids, which increased by increasing the drying temperature of SA at lower acidification pH. The relative amount of diacids increased with an increase in the acidification pH. SEC-RID analysis showed that the most abundant detected fraction was the lower molecular weight fraction with <1300 Da. When comparing different acidification pH, it can be seen that as the pH increased, the lower molecular weight fraction (<1300 Da) slightly decreased. DSC analysis highlighted that elevated sample drying temperature had a higher impact on samples with lower acidification pH values where further SA depolymerization occurred in the sample while drying.

The synthesized SA bio-polyols had high hydroxyl values, which is typical and necessary for polyols to be used for rigid PU foam production. However, the apparent viscosity of bio-polyols was too high. Therefore, in future, if these polyols are to be used in the production of rigid PU foams, further studies need to be conducted to achieve lower acid values and apparent viscosity. Probably a change in the multifunctional alcohol as well as conditions of synthesis or even some extra step such as the oxyalkylation reaction is necessary to obtain SA bio-polyols with the required properties.

## Figures and Tables

**Table 1:** Quantities of the solids, TPC, and hexoses in rinsing water of SA samples.

Sample ^1^	Total Solids, %	TPC, %	Hexoses, %
pH1-0	29.2 ± 0.8	0.21 ± 0.09	3.41 ± 0.23
pH1-1	2.6 ± 0.2	0.04 ± 0.01	0.22 ± 0.04
pH1-2	1.5 ± 0.2	0.05 ± 0.01	0.15 ± 0.01
pH3-0	25.3 ± 0.5	0.22 ± 0.08	1.96 ± 0.27
pH3-1	2.1 ± 0.3	0.04 ± 0.01	0.15 ± 0.02
pH3-2	3.1 ± 0.2	0.10 ± 0.02	0.16 ± 0.03
pH5-0	25.8 ± 0.6	0.49 ± 0.21	1.84 ± 0.12
pH5-1	2.9 ± 0.3	0.08 ± 0.01	0.19 ± 0.06
pH5-2	2.4 ± 0.2	0.10 ± 0.01	0.21 ± 0.04

^1^ Acidification pH—times of rinsing; ± standard deviation.

**Table 2:** Chemical properties of SA samples.

Sample ^1^	TPC, %	Acid Number, mmol/g	Epoxy Groups, mmol/g	Saponification Number, mmol/g	Hydroxyl Number, mmol/g	Dry Matter, %	Yield ^2^, %
pH1-1-50	4.00 ± 0.40	1.76 ± 0.12	0.36 ± 0.01	3.52 ± 0.04	3.90 ± 0.03	36.6	35.3
pH1-1-130	3.81 ± 0.09	1.56 ± 0.03	0.26 ± 0.05	2.85 ± 0.07	3.64 ± 0.01		
pH1-2-50	3.57 ± 0.14	1.84 ± 0.13	0.08 ± 0.02	3.65 ± 0.04	4.09 ± 0.06	36.9	35.6
pH1-2-130	3.90 ± 0.30	1.70 ± 0.02	0.07 ± 0.02	3.72 ± 0.12	3.96 ± 0.04		
pH3-1-50	3.07 ± 0.08	1.78 ± 0.14	0.15 ± 0.01	2.83 ± 0.12	4.08 ± 0.03	27.7	39.0
pH3-1-130	3.00 ± 0.30	1.45 ± 0.04	0.46 ± 0.01	2.85 ± 0.07	3.90 ± 0.06		
pH3-2-50	2.99 ± 0.19	1.44 ± 0.06	0.44 ± 0.03	2.90 ± 0.02	4.30 ± 0.04	27.5	38.7
pH3-2-130	2.18 ± 0.15	1.59 ± 0.05	0.13 ± 0.02	2.65 ± 0.03	4.00 ± 0.03		
pH5-1-50	3.00 ± 0.04	1.46 ± 0.11	0.39 ± 0.03	2.87 ± 0.05	4.43 ± 0.04	26.5	38.9
pH5-1-130	2.19 ± 0.10	1.44 ± 0.02	0.34 ± 0.04	2.78 ± 0.07	3.97 ± 0.06		
pH5-2-50	2.80 ± 0.09	1.90 ± 0.13	0.72 ± 0.01	2.54 ± 0.04	4.43 ± 0.05	26.1	38.2
pH5-2-130	2.29 ± 0.11	1.43 ± 0.05	0.24 ± 0.01	2.37 ± 0.03	4.64 ± 0.04		

^1^ Acidification pH—times of rinsing—drying temperature; ± standard deviation. ^2^ Yield of SA was calculated on the dry mass of the raw material (birch outer bark).

**Table 3:** Main monomers identified by GC-MS analysis of SA samples, before and after hydrolysis (area, rel% from chromatogram).

Identification	pH1-1-50		pH1-1-130		pH3-1-50		pH3-1-130		pH5-1-50		pH5-1-130	
	Met. 1	Met. 2	Met. 1	Met. 2	Met. 1	Met. 2	Met. 1	Met. 2	Met. 1	Met. 2	Met. 1	Met. 2
Alkan-1-ols (total)	-	0.04	0.67	0.01	-	0.06	-	-	-	0.01	-	-
2-undecen-1-ol	-	0.04	0.67	0.01	-	0.06	-	-	-	0.01	-	-
Alkanoic acid (total)	12.38	-	8.80	5.02	-	-	14.85	-	-	-	16.12	-
Octanoic acid	4.22	-	-	-	-	-	-	-	-	-	-	-
5,8,11-eicosatryiynoic acid	-	-	-	0.11	-	-	-	-	-	-	-	-
9,12-octadecadienoic acid	8.16	-	8.80	4.91	-	-	14.85	-	-	-	16.12	-
Hydroxy acids (total)	29.16	89.86	45.54	92.75	38.14	92.54	34.22	89.13	32.87	95.75	29.77	75.86
2-hydroxydecanedioic acid	21.28	89.86	37.03	92.74	24.77	92.54	24.01	89.13	22.57	95.75	20.83	75.86
3-hydroxyhexadecanoic acid	0.63	-	0.33	-	0.47	-	0.44	-	0.43	-	0.33	-
20-hydroxyicosanoic acid	2.55	-	1.91	-	3.15	-	2.27	-	2.56	-	2.31	-
22-hydroxydocosanoic acid	4.7	-	6.27	0.01	9.75	-	7.50	-	7.31	-	6.30	-
Diacids (total)	10.48	5.67	9.66	4.91	17.67	4.79	6.96	4.79	23.27	3.16	5.00	6.56
Octanedioic acid	1.18	-	-	-	-	-	-	-	-	-	-	-
Nonanedioic acid	-	-	-	-	0.31	-	-	-	-	-	-	-
Pentanedioic acid	5.42	-	6.66	-	-	-	3.59	-	1.40	-	0.94	-
Hexadecanedioic acid	1.44	5.67	0.73	-	-	4.79	0.57	4.79	0.66	3.16	0.64	6.56
Decanedioic acid	-	-	-	-	13.10	-	-	-	18.13	-	-	-
10,12-ocosadiynedioic acid	2.44	-	1.64	-	3.18	-	2.14	-	2.37	-	2.69	-
Octadecanedioic acid	-	-	0.63	-	1.08	-	0.66	-	0.71	-	0.73	-
Aromatics (total)	5.97	-	4.90	-	5.86	-	6.14	-	3.88	0.01	1.36	-
Vanilic acid	0.25	-	-	-	-	-	-	-	-	-	-	-
Isoferulic acid	4.83	-	4.90	-	4.39	-	5.04	-	3.88	0.01	1.36	-
Oxyisoflavone	0.89	-	-	-	1.47	-	1.10	-	-	-	-	-
Extractives (total)	39.47	4.44	31.21	2.22	32.31	4.23	36.75	6.07	38.77	1.08	46.2	17.58
Allocholic acid	-	0.21	-	-	-	-	-	-	1.34	-	1.11	-
Lupeol	3.52	4.23	2.96	2.22	2.57	4.23	2.64	6.07	2.19	1.08	2.03	17.58
Betulin	35.95	-	28.25	-	29.74	-	34.11	-	35.24	-	43.06	-
Glycerol derivatives (total)	0.83	-	0.52	-	1.37	-	1.09	-	1.20	-	1.11	-
2-oleoylglycerol	0.83	-	0.52	-	1.37	-	1.09	-	1.20	-	1.11	-
Others (total)	1.73	-	0.62	-	-	-	-	-	-	-	0.43	-
Terephthalic acid	1.73	-	0.62	-	-	-	-	-	-	-	0.43	-

**Table 4:** Relative abundance of high molecular weight compounds in the samples.

A Peak in SEC-RID Chromatogram			Area, rel% from a Chromatogram					
No.	t_R_, min	Mw, Da	pH1-130	pH1-50	pH3-130	pH3-50	pH5-130	pH5-50
1	<7.5	>5500	n	n	n	n	0.1	0.2
2	7.6	4900	0.2	n	0.3	0.3	0.3	0.2
3	7.7	4200	0.4	0.5	0.8	0.8	1.1	1.0
4	7.8	3400	1.6	1.9	2.5	2.7	3.2	3.0
5	8.0	2700	5.4	5.9	6.9	7.3	7.5	8.2
6	8.2	1900	15	15	17	18	17	19
7	>8.4	<1300	77	77	72	71	71	68

**Table 5:** Glass transition (T_g_), melting (T_m1_, T_m2_, T_m3_), and crystallization (T_c_) temperatures of SA from DSC thermograms.

Sample	T_g_, °C	T_m1_, °C	T_m2_, °C	T_m3_, °C	T_c_, °C
pH5-1-50	−22.4	39.6	61.2	73.2	50.2
pH5-1-130	−19.6	41.1	70.4	-	45.7
pH3-1-50	−22.7	39.8	69.8	79.2	37.7
pH3-1-130	−17.9	43.2	51.7	-	26.2
pH1-1-50	−22.1	36.8	71.1	82.2	34.4
pH1-1-130	−18.8	48.4	55.8	-	19.9

**Table 6:** The key characteristics of synthesized bio-polyols.

Polyol	Acid Value, mg KOH/g	Hydroxyl Value, mg KOH/g	Apparent Viscosity at 25 °C (Shear Rate 50 s^−1^), mPa·s	Moisture, %
SA/DEG 1:1.00	26 ± 2	301 ± 3	82,560	0.455 ± 0.011
SA/DEG 1:1.15	21 ± 2	333 ± 5	70,900	0.255 ± 0.016
SA/DEG 1:2.30	12 ± 2	450 ± 5	42,330	0.145 ± 0.001
SA/DEG 1:4.00	5 ± 1	596 ± 4	15,330	0.269 ± 0.013

## Data Availability

Not applicable.
